# Supplementary material for: Self-assembly of sustainable plant protein protofilaments into a hydrogel for ultra-low friction across length scales
Source: Commun Mater. 2024 Sep 3;5(1):158. doi: 10.1038/s43246-024-00590-5 (PMC11371639; doi:10.1038/s43246-024-00590-5)
Supplement: Supplementary file 2 — Supplementary Information [file 43246_2024_590_MOESM2_ESM.pdf]

Supplementary Information

Self-assembly of sustainable plant protein protofilaments into a hydrogel providing ultra-low friction across length scales

Olivia Pabois[a†], Yihui Dong[b†], Nir Kampf[b], Christian D. Lorenz[c], James Doutch[d], Alejandro Avila-Sierra[e], Marco Ramaioli[e], Mingduo Mu[a], Yasmin Message[a], Evangelos Liamas[a,f], Arwen I. I. Tyler[a], Jacob Klein[b], Anwesha Sarkar[a]*

[a] School of Food Science and Nutrition, University of Leeds, Leeds LS2 9JT, United Kingdom

[b] Department of Molecular Chemistry and Materials Science, Weizmann Institute of Science, Rehovot 76100, Israel

[c] Department of Engineering, King's College London, London WC2R 2LS, United Kingdom

[d] ISIS Neutron and Muon Source, Science and Technology Facilities Council, Rutherford Appleton Laboratory, Didcot OX11 ODE, United Kingdom

[e] Université Paris-Saclay, INRAE, AgroParisTech, UMR SayFood, 91120 Palaiseau, France

[f] Unilever Research & Development Port Sunlight, Quarry Road East, Bebington, Merseyside, CH63 3JW, UK

E-mail addresses:
olivia.pabois@gmail.com; yihui.dong@weizmann.ac.il; nir.kampf@weizmann.ac.il; chris.lorenz@kcl.ac.uk; james.doutch@stfc.ac.uk; alejandro.avila-sierra@inrae.fr; marco.ramaioli@inrae.fr; mingduo.mu@mail.mcgill.ca; ymessage@hotmail.co.uk; evangelos.liamas@unilever.com; a.i.i.tyler@leeds.ac.uk; jacob.klein@weizmann.ac.il; a.sarkar@leeds.ac.uk

* Corresponding author:
Prof. Anwesha Sarkar
University of Leeds
School of Food Science and Nutrition, LS2 9JT Leeds, UK
Email: a.sarkar@leeds.ac.uk

[†] Authors contributed equally to this work

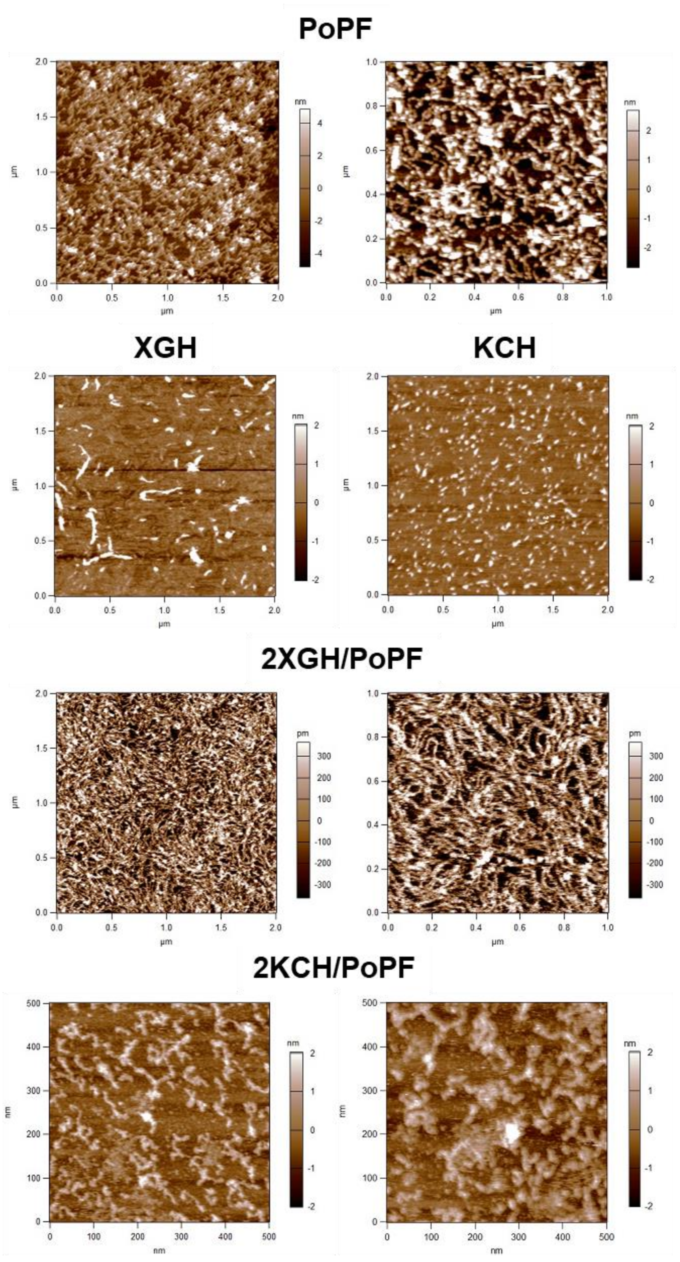

Supplementary Figure 1| Nanostructure of the self-assembled protofilament/hydrogel formed using different polysaccharides. Atomic force microscopy (AFM) images of the self-assesmbled protofilament/hydrogel fabricated using different polysaccharide types (either xanthan gum (XG) or *κ*-carrageenan (KC)), and each of their individual components, following sample deposition onto a negatively charged, hydrophilic (mica) surface and immersion in citrate buffer (pH 3.0). While PoPF seems to form amyloid-like protofilaments, the nanostructure of none of the polysaccharide hydrogels (either XGH or KCH) could be visualised by AFM due to their lack of interaction with the mica surface. XGH seems to form a continuous polymeric network in which PoPF protofilaments are uniformly distributed, whereas KCH seems to interact with PoPF forming interconnected clusters.

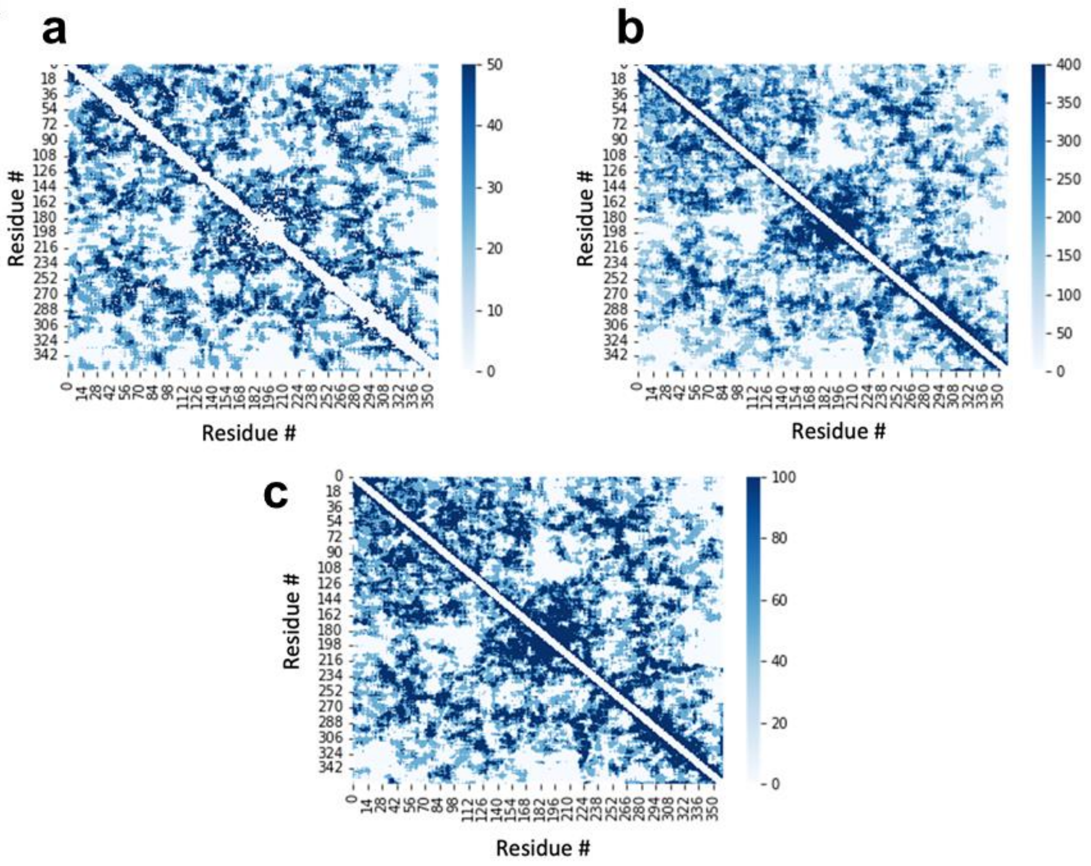

Supplementary Figure 2| Contact maps of potato protein (patatin) within the potato protein protofilaments (PoPF) and self-assembly (2 XGH/PoPF). Contact maps for the patatins within **a|** the simulated PoPF in an aqueous environment, **b|** the simulated PoPF in a xanthan gum hydrogel (XGH) aqueous environment (2 XGH/PoPF), and **c|** the simulated PoPF in a XGH aqueous environment (2 XGH/PoPF) in the presence of a PDMS substrate. Contacts are formed by residues whose centres of mass are within 1.5 nm of one another. As the contacts are generally the same in all systems, the internal structure of these PoPF does not change significantly.

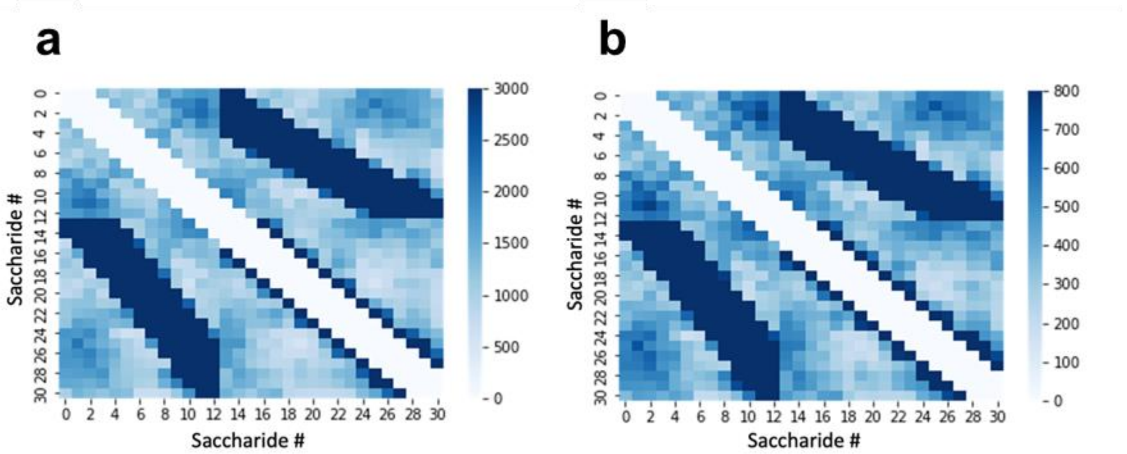

Supplementary Figure 3| Contact maps of xanthan gum (XG) molecules coating the potato protein protofilaments (PoPF) in the self-assembled protofilament/hydrogel system (2 XGH/PoPF). Contact maps for the XG molecules within the simulated 2 XGH/PoPF system in **a|** an aqueous environment and **b|** in the presence of a PDMS substrate. Contacts are formed by saccharides whose centres of mass are within 1.5 nm of one another. As the contacts are generally the same in all systems, the nature of the interactions of the xanthan gum molecules is not affected by the presence of the PDMS substrate.

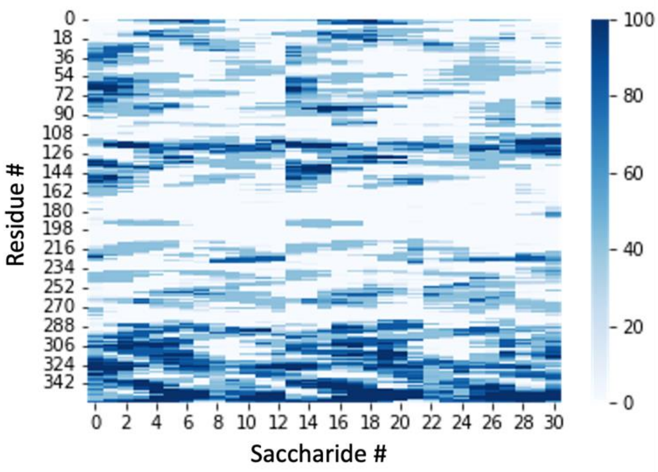

Supplementary Figure 4| Contact map of potato protein (patatin) and xanthan gum (XG) molecules within the self-assembled protofilament/hydrogel system (2 XGH/PoPF). Contacts are formed between the aminoacids in patatin and the saccharides in XG whose centres of mass are within 1.5 nm of one another.

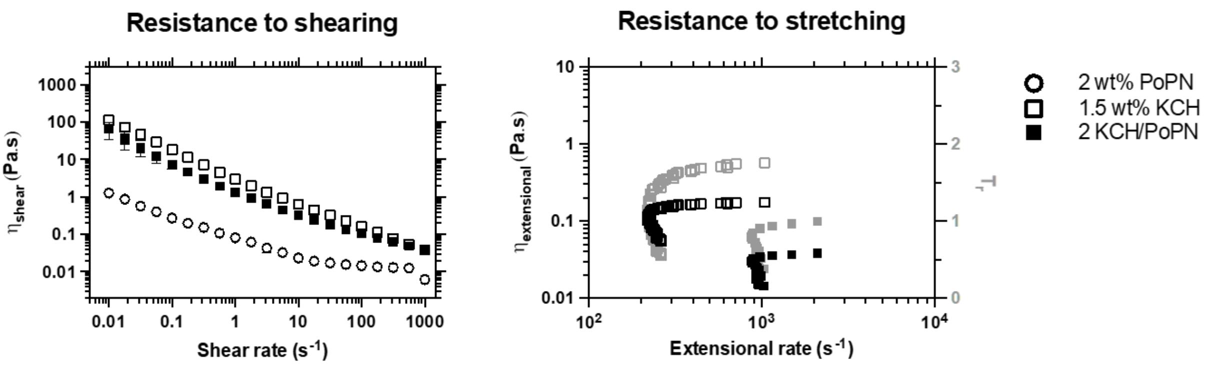

Supplementary Figure 5| Viscoelastic behaviour of the self-assembled protofilament/hydrogel using a different polysaccharide. Evolution of **a|** the shear viscosity (η_{shear}) as a function of the shear rate, obtained from stress-controlled rotational rheometry measurements, and **b|** the extensional viscosity ($\eta_{extensional}$) and Trouton ratio (T_r) as a function of the extensional rate, obtained from extensional rheometry measurements, performed on the self-assembled potato protein protofilament/hydrogel fabricated using a different polysaccharide type (2 KCH/PoPF) and each individual component (1.2 wt% KCH, and 0.6 wt% PoPF), at 37 °C. Similarly to 2 XGH/PoPF (**Figure 3**), the self-assembled protofilament/hydrogel system made with KCH (2 KCH/PoPF) behaves like the polysaccharide hydrogel (KCH) on its own in term of shear rheology, displaying both high shear viscosity values (η_{shear} = 67 ± 32 Pa.s for 2 KCH/PoPF *vs.* η_{shear} = 111 ± 48 Pa.s for KCH, at 0.01 s^{-1}) and a notable shear rate-dependent decrease (up to η_{shear} = 0.04 ± 0.01 Pa.s for 2 KCH/PoPF *vs.* η_{shear} = 0.039 ± 0.003 Pa.s for KCH, at 1,000 s^{-1}), but shows discrepancies in term of extensional rheology, exhibiting much lower extensional viscosity (ca. $\eta_{extensional}$ = 0.80 ± 0.4 Pa.s for 2 KCH/PoPF *vs.* ca. $\eta_{extensional}$ = 5.8 ± 0.9 Pa.s for KCH) and Trouton ratio (ca. T_r = 9.7 ± 4.1 for 2 KCH/PoPF *vs.* ca. T_r = 58.7 ± 39.2 for KCH) values. Compared to 2 XGH/PoPF (**Figure 3**), 2 KCH/PoPF displays lower shear and extensional viscosity values, as well as a lower Trouton ratio. The extensional rheology of PoPF could not be assessed due to its too short breaking time (ca. t_b = 0.014 s). Each experiment was reproduced at least three times; the average and a representative measurement are shown for shear and extensional rheology, respectively. The notable variation observed among samples, as indicated by the standard deviation in extensional measurements, can be attributed to the heterogeneous composition of the formulations.

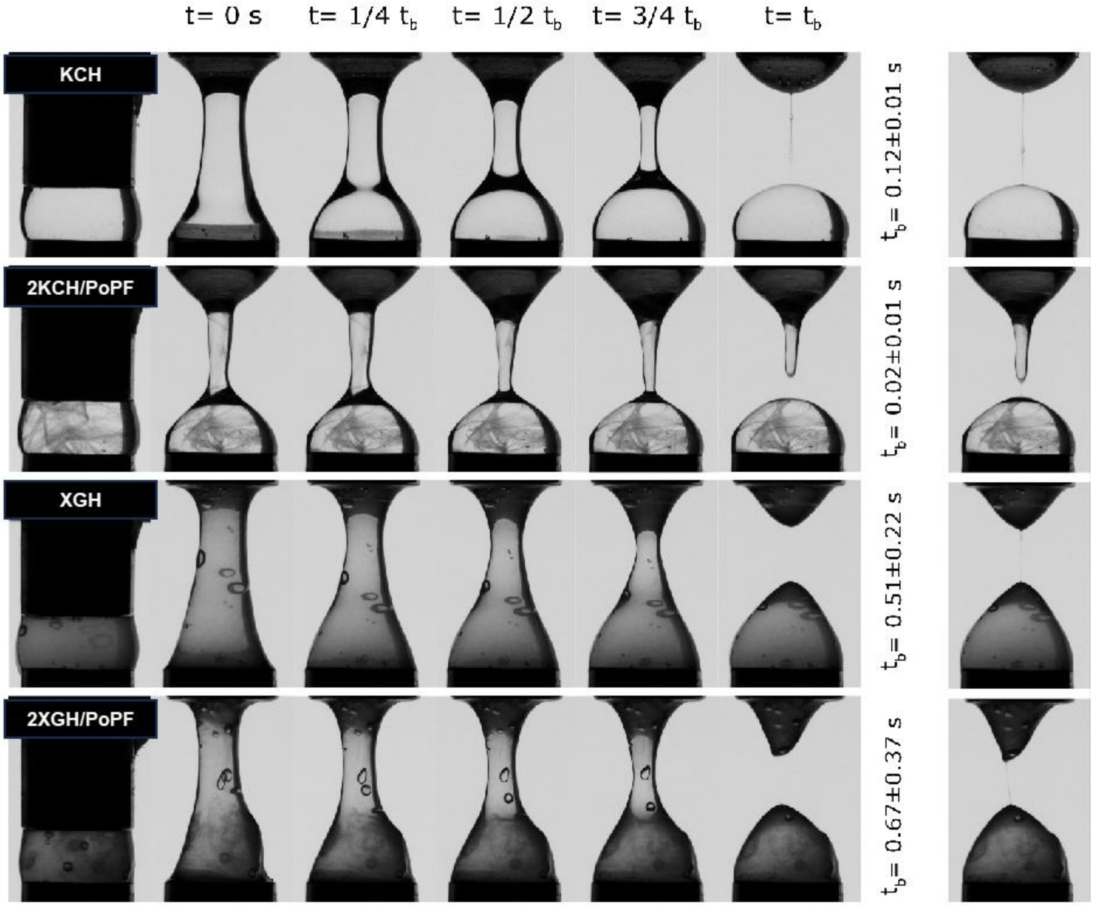

Supplementary Figure 6| Capillary thinning properties of the self-assembled protofilament/hydrogel fabricated employing different polysaccharide types. Stretching-induced capillary thinning behaviour observed over time with an extensional rheometer, for the self-assembled potato protein protofilament/hydrogel fabricated using different polysaccharide types forming hydrogels (either XGH or KCH), at 37°C. Times normalised with respect to the capillary break-up time (t_b) are indicated, and an extra column on the right shows the shape of the filament just before break-up. PoPF is not shown here as the filament broke before reaching the final axial displacement imposed. The 2 XGH/PoPF self-assembled protofilament/hydrogel filament breaks at $t_b = 0.67 \pm 0.37$ s, thus being more resistant to thread thinning than XGH for which $t_b = 0.51 \pm 0.22$ s. Contrary to 2 XGH/PoPF, 2 KCH/PoPF exhibits a shorter breaking time than KCH ($t_b = 0.02 \pm 0.01$ s for 2 KCH/PoPF *vs*. $t_b = 0.12 \pm 0.01$ s for KCH). Compared to 2 XGH/PoPF, 2 KCH/PoPF displays a lower resistance to capillary thinning. Each experiment was reproduced at least three times; a representative measurement is shown.

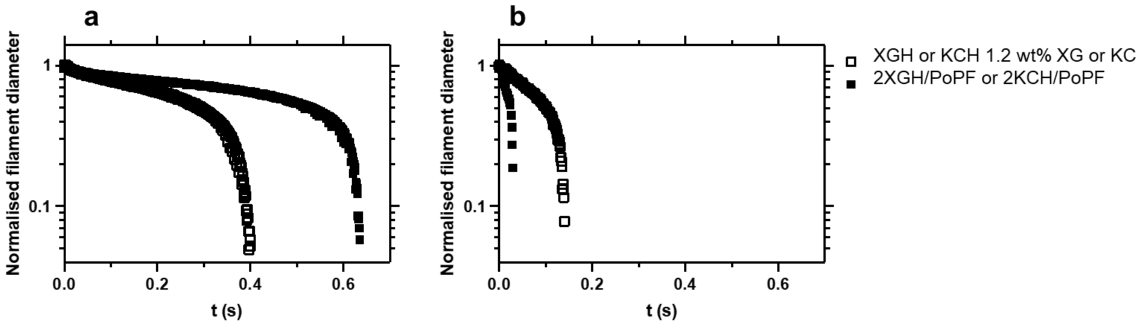

Supplementary Figure 7| Normalised filament diameter of the self-assembled protofilament/hydrogel fabricated employing different polysaccharide types and of each polysaccharide used on their own. Time-dependent evolution of the normalised filament diameter upon stretching, measured by extensional rheometry, at 37°C, for the self-assembled potato protein protofilament/hydrogel fabricated using **a|** xanthan gum hydrogel (XGH) and **b|** *κ*-carrageenan hydrogel (KCH). PoPF is not shown here as the filament broke before reaching the final axial displacement imposed. All samples exhibit a two-stage filament thinning mechanism, with (i) an initial, exponential regime, where a long thread forms, (ii) followed by a fast and marked exponential decay rapidly evolving into an axially uniform thin filament, eventually breaking up. 2 XGH/PoPF shows a higher resistance to capillary thinning than XGH, oppositely to 2 KCH/PoPF. Additionally, the filament formed by 2 XGH/PoPF seems to break up much later than the one formed by 2 KCH/PoPF. Each experiment was reproduced at least three times; a representative measurement is shown.

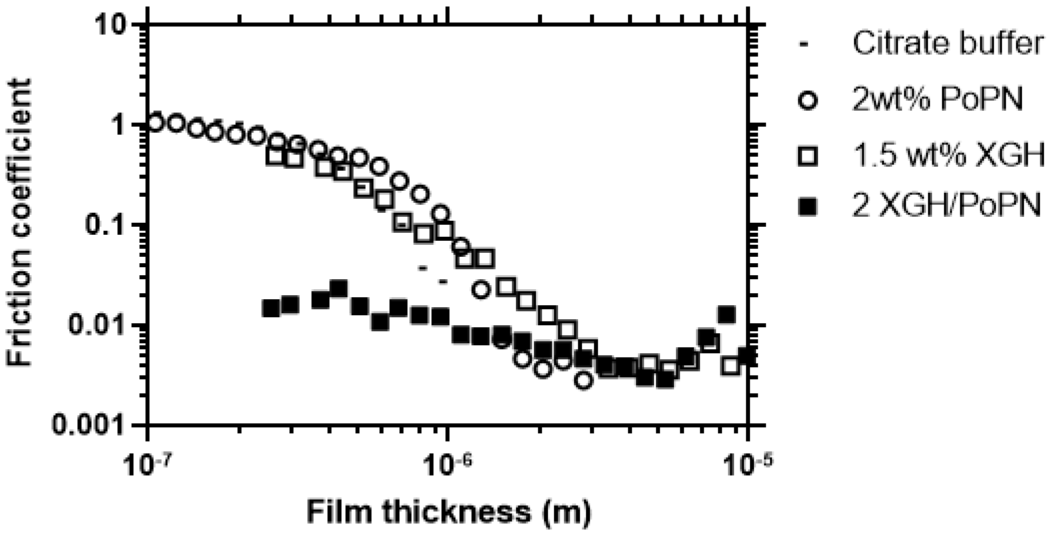

Supplementary Figure 8| Macroscale lubrication performance of the self-assembled protofilament/hydrogel as a function of film thickness. a| Evolution of the friction coefficient as a function of mimimum elastohydrodynamic film thickness (h_{min}), obtained from tribology measurements and rheology measurements at 37 °C. The lubrication properties of citrate buffer are also shown for comparison purposes. Whilst all other curves overlap, 2 XGH/PoPF shows an outstanding lubrication performance both in the boundary and hydrodynamic regions, exhibiting ultra-low friction coefficients contrary to both PoPF and XGH on their own. Each measurement was reproduced at least three times; the average measurement is shown.

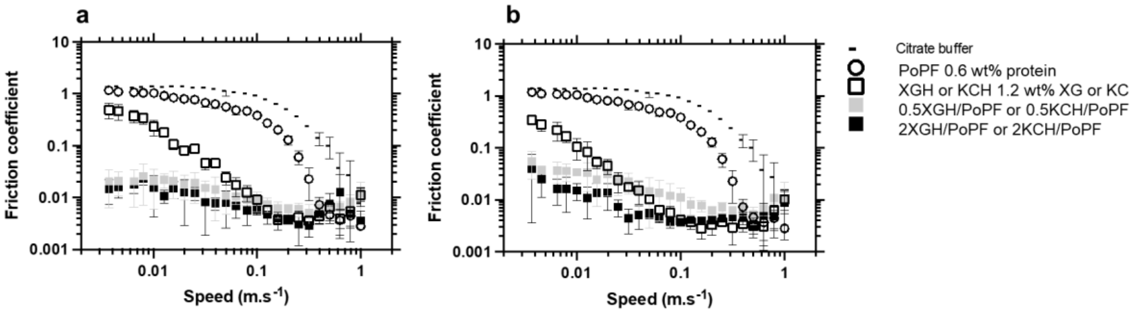

Supplementary Figure 9| Macroscale lubrication performance of the self-assembled protofilament/hydrogel made using different polysaccharide/protein ratios and different polysaccharide types. Speed-dependent evolution of the friction coefficient, obtained from tribology measurements performed with non-charged, hydrophobic (PDMS) surfaces (at the macroscale), on the self-assembled potato protein protofilament/hydrogel fabricated using different polysaccharide types: **a|** xanthan gum hydrogel (XGH) and **b|** *κ*-carrageenan hydrogel (KCH), as well as different polysaccharide/protein ratios (either 0.5 or 2, corresponding to a mixture of either 1.0 wt%/2.0 wt% or 1.2 wt%/0.6 wt% polysaccharide/protein), at 37 °C. The lubrication properties of each individual component and citrate buffer at pH 3.0 are also shown for comparison purposes. Independently of the polysaccharide/protein ratio and polysaccharide type used in this study, the fabricated self-assembled protofilament/hydrogel demonstrates ultra-low friction both in the boundary and hydrodynamic regions, exhibiting much lower friction coefficients than the sole components (*i.e.,)*. Each measurement was reproduced at least three times; the average measurement is shown.

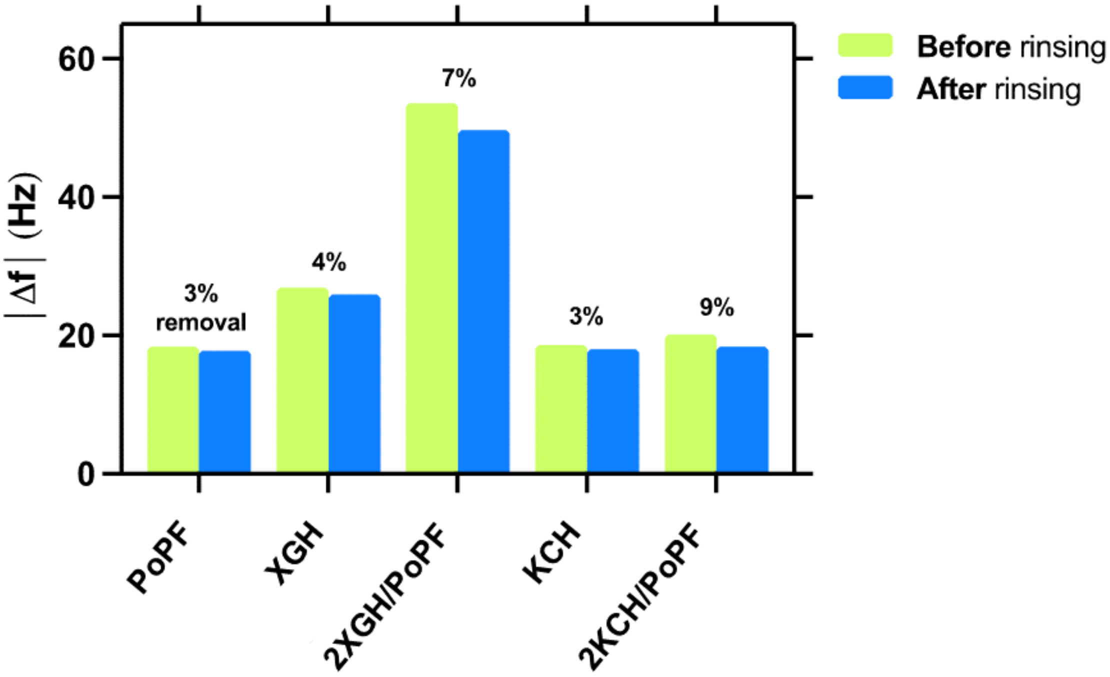

Supplementary Figure 10| Adsorption capacity of the self-assembled protofilament/hydrogel made using different polysaccharide types, in the presence of a PDMS-coated surface. Comparison of the resonance frequency (Δ*f*) reached before and after rinsing, which is an indication of the adsorption extent, obtained for the self-assembled protofilament/hydrogel fabricated using different polysaccharide types (xanthan gum hydrogel (XGH) and κ-carrageenan hydrogel (KCH)), and each individual component (1.2 wt% XGH or KCH, and 0.6 wt% PoPF), in the presence of a PDMS-coated surface. The proportion of material removed, or the desorption extent, is also indicated and calculated considering the decrease in resonance frequency (Δ*f*) following rinsing; this parameter is used here as an *in vitro* indicator of the coating index of the samples, higher values corresponding to lower coating indices. These data were extracted from the QCM-D measurements (Figure 4c). Each sample was diluted 20 times prior to any measurements. The self-assembled potato protein protofilament/hydrogel fabricated using XGH (2 XGH/PoPF) readily adsorbs at the interface, to a much higher extent than PoPN and XGH on their own, and both systems (2 XGH/PoPF and 2 KCH/PoPF) remain strongly attached following rinsing, losing only 7% and 9% of its adsorbed layer, respectively. Each measurement was reproduced at least three times; the average measurement is shown.

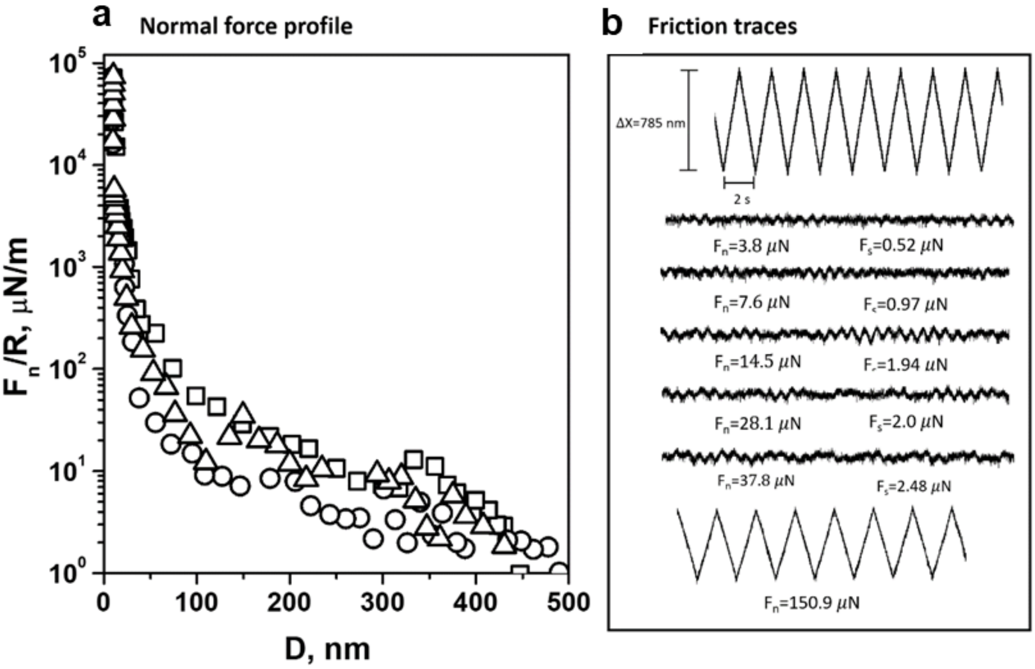

Supplementary Figure 11| Nanoscale friction for citrate buffer at pH 3 measured by surface force balance (SFB). a| Normal force (F_n), normalised by the radius of curvature *vs*. surface separation distance (*D*) between the curved mica surfaces (F_n/R *vs*. *D*) across the citrate buffer solution at pH 3.0. Different symbols represent the different contact positions on one pair of mica. **b|** Typical traces of shear force *vs*. time for sliding mica surfaces across the citrate buffer solution at pH 3.0.

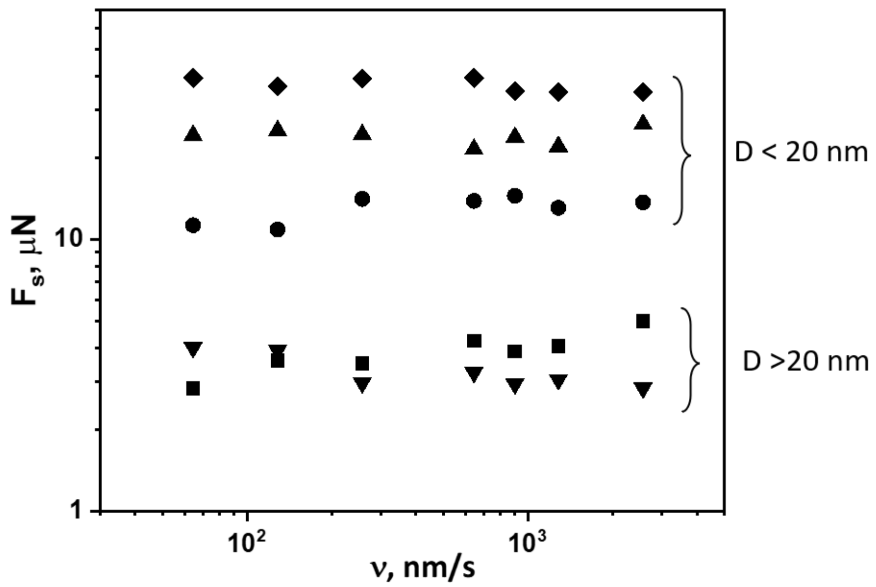

Supplementary Figure 12| Nanoscale friction for xanthan gum hydrogel (XGH) measured by surface force balance (SFB). Representative shear force (F_s) as a function of sliding velocity (v) between the mica surfaces across the xanthan gum hydrogel (XGH)dispersion, at a surface separation distance D > 20 nm and < 20 nm. The sliding velocity was adjusted by changing the frequency at a fixed lateral displacement amplitude.

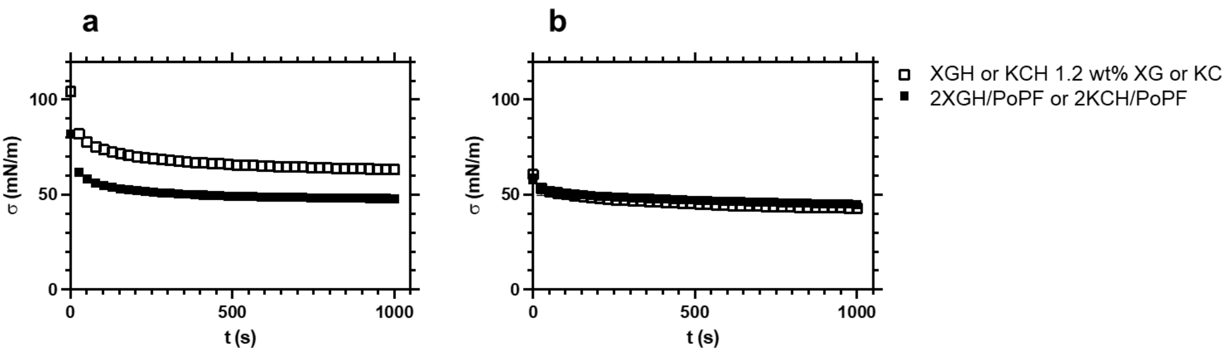

Supplementary Figure 13| Surface tension of the self-assembled protofilament/hydrogel fabricated employing different polysaccharide types. Time-dependent evolution of the surface tension (*σ*) of the self-assembled potato protein protofilament (PoPF)/hydrogel fabricated using different polysaccharide types: **a|** xanthan gum hydrogel (XGH), and **b|** *κ*-carrageenan hydrogel (KCH), measured with a Wilhelmy plate tensiometer, at 37 °C. PoPF is not shown here as the filament broke before reaching the final axial displacement imposed. 2 XGH/PoPF is more surface active than XGH (*i.e.*, $\sigma = 47.9 \pm 1.0$ mN.m^{-1} for 2 XGH/PoPF *vs*. $\sigma = 63.3 \pm 1.7$ mN.m^{-1} for XGH), whilst 2 KCH/PoPF behaves similarly to KCH (*i.e.*, $\sigma = 44.9 \pm 1.6$ mN.m^{-1} for 2 KCH/PoPN *vs*. $\sigma = 42.8 \pm 0.5$ mN.m^{-1} for KCH). Each measurement was reproduced at least three times; the average measurement is shown.

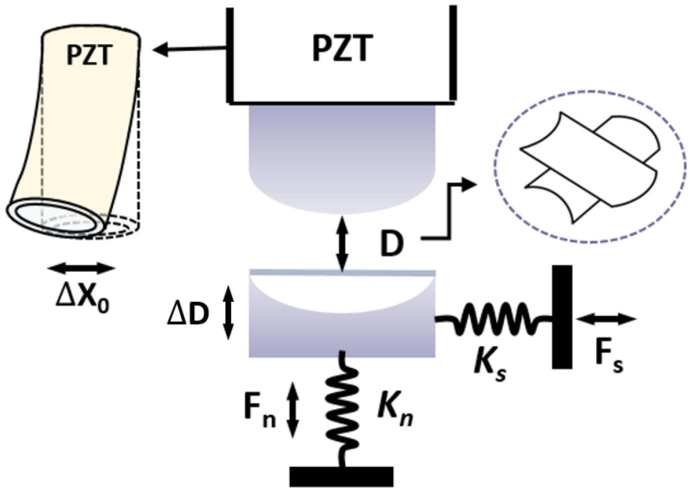

Supplementary Figure 14| Schematic illustration of the surface force balance (SFB) setup. The two back-silvered mica sheets are glued on two cylindrical quartz lenses separately in a crossed-cylinder configuration (in the dashed circle). The top lens with its mica surface is mounted on a 4-sectored piezoelectric tube to which a lateral motion Δx_0 may be applied, while the bottom lens with its mica surface is mounted on the normal force spring. *D* represents the separation between two mica surfaces. K_n and K_s are the normal and shear force springs, respectively. The normal (F_n) and shear (F_s) forces are evaluated respectively from the bending of these two orthogonal springs.
